# The molecular portraits of breast tumors are conserved across microarray platforms

**DOI:** 10.1186/1471-2164-7-96

**Published:** 2006-04-27

**Authors:** Zhiyuan Hu, Cheng Fan, Daniel S Oh, JS Marron, Xiaping He, Bahjat F Qaqish, Chad Livasy, Lisa A Carey, Evangeline Reynolds, Lynn Dressler, Andrew Nobel, Joel Parker, Matthew G Ewend, Lynda R Sawyer, Junyuan Wu, Yudong Liu, Rita Nanda, Maria Tretiakova, Alejandra Ruiz Orrico, Donna Dreher, Juan P Palazzo, Laurent Perreard, Edward Nelson, Mary Mone, Heidi Hansen, Michael Mullins, John F Quackenbush, Matthew J Ellis, Olufunmilayo I Olopade, Philip S Bernard, Charles M Perou

**Affiliations:** 1 Lineberger Comprehensive Cancer Center, University of North Carolina, Chapel Hill, NC 27599, USA; 2 Department of Genetics, University of North Carolina, Chapel Hill, NC 27599, USA; 3 Department of Statistics and Operations Research, University of North Carolina, Chapel Hill, NC 27599, USA; 4 Department of Biostatistics, University of North Carolina, Chapel Hill, NC 27599, USA; 5 Department of Pathology and Laboratory Medicine, University of North Carolina, Chapel Hill, NC 27599, USA; 6 Department of Medicine, University of North Carolina, Chapel Hill, NC 27599, USA; 7 Constella Health Sciences, 2605 Meridian Parkway, Durham, NC 27713, USA; 8 Section of Hematology/Oncology, Department of Medicine, Committees on Genetics and Cancer Biology, University of Chicago, 5841 South Maryland Avenue, Chicago, IL 60637-1463, USA; 9 Department of Pathology, Thomas Jefferson University, 132 South 10th Street Philadelphia, PA 19107, USA; 10 The ARUP Institute for Clinical and Experimental Pathology, 500 Chipeta Way, Salt Lake City, Utah 84108, USA; 11 Department of Surgery, University of Utah School of Medicine, 30 N 1900 E, Salt Lake City, Utah 84132, USA; 12 Department of Pathology, University of Utah School of Medicine, 30 N 1900 E, Salt Lake City, Utah 84132, USA; 13 Department of Medicine, Division of Oncology, Washington University School of Medicine and Siteman Cancer Center, St Louis, Missouri, USA

## Abstract

**Background:**

Validation of a novel gene expression signature in independent data sets is a critical step in the development of a clinically useful test for cancer patient risk-stratification. However, validation is often unconvincing because the size of the test set is typically small. To overcome this problem we used publicly available breast cancer gene expression data sets and a novel approach to data fusion, in order to validate a new breast tumor intrinsic list.

**Results:**

A 105-tumor training set containing 26 sample pairs was used to derive a new breast tumor intrinsic gene list. This intrinsic list contained 1300 genes and a proliferation signature that was not present in previous breast intrinsic gene sets. We tested this list as a survival predictor on a data set of 311 tumors compiled from three independent microarray studies that were fused into a single data set using Distance Weighted Discrimination. When the new intrinsic gene set was used to hierarchically cluster this combined test set, tumors were grouped into LumA, LumB, Basal-like, HER2+/ER-, and Normal Breast-like tumor subtypes that we demonstrated in previous datasets. These subtypes were associated with significant differences in Relapse-Free and Overall Survival. Multivariate Cox analysis of the combined test set showed that the intrinsic subtype classifications added significant prognostic information that was independent of standard clinical predictors. From the combined test set, we developed an objective and unchanging classifier based upon five intrinsic subtype mean expression profiles (i.e. centroids), which is designed for single sample predictions (SSP). The SSP approach was applied to two additional independent data sets and consistently predicted survival in both systemically treated and untreated patient groups.

**Conclusion:**

This study validates the "breast tumor intrinsic" subtype classification as an objective means of tumor classification that should be translated into a clinical assay for further retrospective and prospective validation. In addition, our method of combining existing data sets can be used to robustly validate the potential clinical value of any new gene expression profile.

## Background

The classification of human tumors using microarray data has been an area of intense research, but it remains a daunting task to validate a new profile and generate a clinically useful test. Many different gene expression-based predictors have been developed for breast cancer [1-9], and two different gene expression predictors have reached the final step of prospective clinical trial testing [10,11]. Using cDNA microarrays, we previously identified five distinct subtypes of breast tumors arising from at least two distinct cell types (basal-like and luminal epithelial cells) [1-3]. This molecular taxonomy was based upon an "intrinsic" gene set, which was identified using a supervised analysis to select genes that showed little variance within repeated samplings of the same tumor, but which showed high variance across tumors [1]. We showed that an intrinsic gene set reflects the stable biological properties of tumors and typically identifies distinct tumor subtypes that have prognostic significance, even though no knowledge of outcome was used to derive this gene set [3,12-14].

A major challenge for microarray studies, especially those with clinical implications, is validation [15,16]. Due to the practical barriers of cost and access to large numbers of fresh frozen tumor samples with associated clinical information, very few microarray studies have analyzed enough samples to allow promising initial findings to be sufficiently validated to justify the major investment required for clinical testing. An efficient approach would be to use public gene expression data repositories as test sets; however, it has been difficult to compare and/or combine data sets from independent laboratories due to differences in sample preparation, experimental design, and microarray platforms. An accepted method for validation is to derive a prognostic/predictive gene set from a "training set" and then apply it to a completely independent "test set" [17]. The "purest" test sets are comprised of samples not generated by the primary investigators to remove any possibility of bias [18]. In this study, we illustrate the successful application of these principles by (1) deriving a new breast tumor intrinsic gene list that identifies the "intrinsic" biological features of breast tumors and (2) validating this predictor using a combined test set of 311 breast tumor samples compiled from the public domain. These analyses show that the breast tumor intrinsic subtypes are significant predictors of outcome when correcting for standard clinical parameters, and that common patterns of expression and outcome predictions can be identified in data sets generated by independent labs.

## Results

### Identification of the Intrinsic/UNC gene set

**Figure 1:**
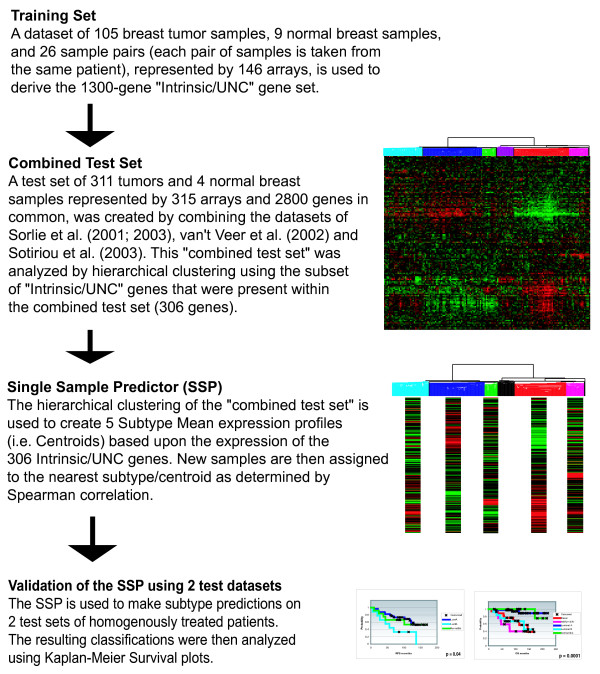
Overview of the analysis methods and datasets used in this paper.

Our goals were to (1) create a new breast tumor intrinsic list, (2) validate this list on an independent dataset to show the clinical significance of the "intrinsic" classifications, and (3) to derive an objective "intrinsic subtype" classifier that could be used clinically (see Figure 1 for overview of analyses performed). An intrinsic analysis is a "within class" versus "across classes" analysis that identifies genes that show low variability within a group (i.e. a tumor-metastasis pair), but which show high variation in expression across different tumors; in essence, one is selecting for genes that are consistently expressed when individual tumors are examined, but that vary in expression across different tumors. To develop a new breast tumor intrinsic gene set (Intrinsic/UNC), we assayed a training set of 105 breast tumor samples and 9 normal breast samples, which contained 26 sample pairs (See Additional file 2, 146 microarray experiments in total), using Agilent oligo microarrays. Using the intrinsic analysis method as described in Sorlie et al. 2003[3], we identified an intrinsic gene set of 1410 microarray elements representing 1300 genes. We felt it important to create a new intrinsic list because first, we wanted to take advantage of newer microarrays (Agilent arrays with 17,000 genes vs. 8,000 gene cDNA microarrays previously used[3]), and second, we wanted to use paired tumor samples that were not before-and-after chemotherapy pairs, but were instead pre-treatment tumor pairs. The Intrinsic/UNC gene set showed overlap with a previous breast tumor intrinsic gene set (108 genes in common with the Intrinsic/Stanford gene set of Sorlie et al. 2003[3]), but also showed a significant increase in gene number likely due to the greater number of genes present on current microarrays.

### Validation of the Intrinsic/UNC gene list

**Figure 2:**
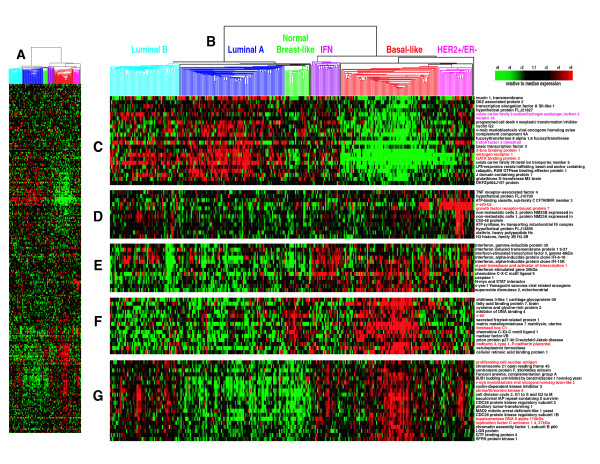
Hierarchical cluster analysis of the 315-sample combined test set using the Intrinsic/UNC gene set reduced to 306 genes. **(A) **Overview of complete cluster diagram. **(B) **Experimental sample-associated dendrogram. **(C) **Luminal/ER+ gene cluster with *GATA3*-regulated genes highlighted in pink. **(D) ***HER2 *and *GRB7*-containing expression cluster. **(E) **Interferon-regulated cluster containing *STAT1*. **(F) **Basal epithelial cluster. **(G) **Proliferation cluster.

To evaluate the Intrinsic/UNC gene set on an independent test dataset, we applied it to a "combined test set" of 315 breast samples (311 tumors and 4 normal breast samples) using hierarchical clustering methods as have been done previously [1-3]. The "combined test set" of 315 breast samples was a single data set created by combining together the data from Sorlie *et al*. 2001 and 2003 (cDNA microarrays)[2,3], van't Veer *et al*. 2002 (custom Agilent oligo microarrays)[5] and Sotiriou *et al.*2003 (cDNA microarrays)[19]. We created a single data table of these three sets by first identifying the common genes present across all three microarray data sets (2800 genes). Next, we used Distance Weighted Discrimination (DWD) to combine these three data sets together [20]. DWD is a multivariate analysis tool that is able to identify systematic biases present in separate data sets and then make a global adjustment to compensate for these biases; in essence, each separate data set is a multi-dimensional cloud of data points, and DWD takes two points clouds and shifts one such that it more optimally overlaps the other. Finally, we determined that 306 of the 1300 unique Intrinsic/UNC genes were present in the combined test set and performed a hierarchical clustering analysis of these 306 genes and 315 samples (Figure 2; see Additional file 1, for the complete cluster diagram). We analyzed the combined test set instead of analyzing each of the 3 datasets separately because we believed this would provide more statistical power to perform multivariate analysis, and would yield more meaningful results because any finding would need to be shared/present across all 3 datasets. Remarkably, despite the loss of genes in the Intrinsic/UNC list due to the requirement of having to be present on 4 different microarray platforms, the hierarchical clustering analysis in Figure 2 identified the five main subtypes/groups corresponding to the previously defined HER2+/ER-, Basal-like, LumA, LumB and Normal Breast-like tumor groups [2,3].

As shown in previous studies, a HER2+ expression cluster was observed in the cluster analysis of the "combined test set" and contained multiple genes from the 17q11 amplicon including *HER2/ERBB2 *and *GRB7 *(Figure 2D). The HER2+ intrinsic subtype (pink dendrogram branch in Figure 2B) was predominantly ER-negative (i.e. HER2+/ER-) as previously shown. A Basal-like expression cluster was also present and contained genes (i.e. *c-KIT, FOXC1 *and *P-Cadherin*) previously identified to be characteristic of basal epithelial cells (Figure 2F). Using the program EASE[21], the Gene Ontology (GO) categories "extracellular space" and "extracellular region" were over-represented relative to chance in the Basal epithelial gene cluster. As shown in previous studies, a Luminal/ER+ expression cluster was present and contained *ER*, *XBP1*, *FOXA1 *and *GATA3 *(Figure 2C). *GATA3 *has recently been shown to be somatically mutated in some ER+ breast tumors, and some of the genes in Figure 2C are *GATA3*-regulated (*FOXA1 *and *TFF3*)[22], thus showing the functional clustering of a transcription factor and some of its direct targets. The Gene Ontology (GO) categories "transcription regulator activity" and "DNA binding" were over-represented relative to chance in the Luminal/ER+ gene cluster.

The most significant difference between the previous Intrinsic/Stanford gene lists and the new Intrinsic/UNC gene list was that the latter contained a large proliferation signature (Figure 2G) [23-25]. As expected, EASE analysis showed that the GO categories "mitotic cell cycle" and "M phase" were over-represented relative to chance in the proliferation signature. The inclusion of proliferation genes in the Intrinsic/UNC gene set, but not in the Intrinsic/Stanford gene set, is likely due to the fact that the Intrinsic/Stanford lists were based upon before-and-after chemotherapy paired samples of the same tumor, while the Intrinsic/UNC list was based upon paired samples taken at the same time point with respect to chemotherapy (22/26 were pre-treatment pairs). This finding suggests that tumor cell proliferation rates do vary before and after chemotherapy, but that proliferation is a reproducible and intrinsic feature of a tumor's expression profile.

A possible new tumor group (IFN) characterized by the high expression of Interferon (IFN)-regulated genes was observed in the combined test set analysis (Figure 2E). According to EASE, the GO categories "immune response" and "defense response" were over-represented relative to chance in the interferon-regulated gene cluster. This cluster contained *STAT1*, which is thought to be the transcription factor responsible for mediating IFN-regulation of gene expression [26,27]. Genes in the IFN cluster have been linked to lymph node metastasis and poor prognosis [7,13]. In summary, the Intrinsic/UNC list contained more genes than previous lists, encompasses most features of the Intrinsic/Stanford list (i.e. Basal, Luminal/ER+, and HER2-amplicon gene clusters) and adds the biologically and clinically relevant proliferation signature.

### Tumor subtypes identified by the Intrinsic/UNC gene set are predictive of outcome

**Figure 3:**
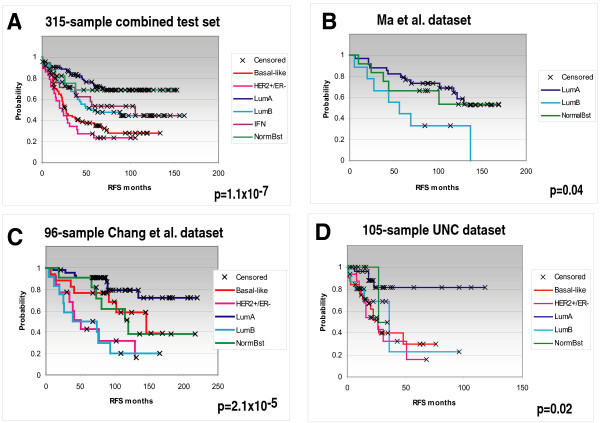
Kaplan-Meier survival curves of breast tumors classified by intrinsic subtype. Survival curves are shown for **(A) **the 315-sample combined test set classified by hierarchical clustering using the Intrinsic/UNC gene set and **(B) **the 60-sample Ma et al., **(C) **96-sample Chang et al., and **(D) **105-sample (used to derive the Intrinsic/UNC gene set) datasets classified by the Nearest-Centroid predictor (Single Sample Predictor).

To determine how many biologically relevant tumor subtypes/groups might be present within the cluster in Figure 2, we used 3 criteria, which resulted in the identification of 6 potential subtypes/groups. The first criterion was the simple and obvious dendrogram branching pattern (Figure 2B) suggesting six groups. Second was the observation that each of the six groups uniquely expressed distinct sets of known biologically relevant genes including the basal, luminal/ER+, HER2-amplicon, IFN-regulated, and proliferation-associated signatures. Third was our knowledge of the previous classifications made by the Sorlie et al. 2003 Intrinsic/Stanford list of the Stanford/Norway samples (these samples are identified in Additional file 1): there was a high concordance (78%) between the classification of these samples made using either the Sorlie et al. 2003 Intrinsic/Stanford list or the Intrinsic/UNC list (excluding the IFN samples). Therefore, the 311 tumors/patients were stratified into six groups, and we proceeded to look for differences in outcomes and associations with other clinical parameters between these six groups. The Intrinsic/UNC gene set identified tumor groups/subtypes that were predictive of Relapse-Free Survival (RFS, Figure 3A) and Overall Survival (OS, p = 0.000001, data not shown) in Kaplan-Meier survival analysis on the combined test set. As previously seen in Sorlie et al. (2001 and 2003), the LumA group had the best outcome while the HER2+/ER-, Basal-like, and LumB groups had significantly worse outcomes. The new IFN class had a Kaplan-Meier survival curve similar to that of LumB, and both showed elevated proliferation rates when compared to LumA (Figure 2G).

**Figure 4:**
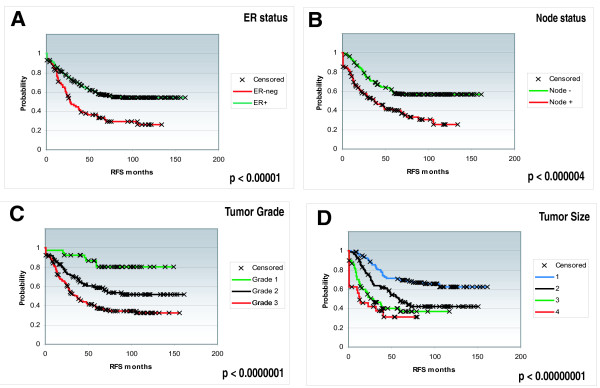
Kaplan-Meier survival curves using RFS as the endpoint, for the common clinical parameters present within the 315-sample combined test set. Survival curves are shown for **(A) **ER status, **(B) **node status, **(C) **histologic grade (1 = well-differentiated, 2 = intermediate, 3 = poor), and **(D) **tumor size (1 = diameter of 2 cm or less; 2 = diameter greater than 2 cm and less than or equal to 5 cm; 3 = diameter greater than 5 cm; 4 = any size with direct extension to chest wall or skin).

In the combined test set, the standard clinical parameters of ER status, node status, grade, and tumor size (note: data for clinical HER2 status was not available) were significant predictors of RFS using Kaplan-Meier analysis (Figure 4), thus showing that the act of combining three different patient sets together did not destroy the prognostic abilities of these standard markers. In a multivariate Cox proportional hazards analysis of the combined test set using these standard clinical parameters, size, grade and ER status were significant predictors of RFS (Table 1A).

To further evaluate the prognostic/predictive value of the intrinsic subtype classification, we performed multivariate Cox proportional hazards analysis of the combined test set using the six intrinsic subtypes/groups defined above and the five standard clinical parameters with RFS, OS, or DSS as the endpoint (Table 1B shows analysis for RFS). The intrinsic subtypes, when added to the multivariate model containing the standard clinical variables, resulted in a model significantly more predictive of RFS, OS, and DSS (p = 0.01, 0.009, and 0.04 respectively, by the likelihood-ratio test). In multivariate analysis for RFS (Table 1B), the Basal-like, LumB and HER2+/ER- subtypes had hazard ratios significantly greater than 1 (LumA served as the reference group), while the IFN and Normal Breast-like groups were not significant. Thus, the intrinsic subtypes classifications of LumA, LumB, Basal-like and HER2+/ER- add new and important prognostic information beyond what the standard clinical predictors provide.

### Associations of the intrinsic subtypes with clinical and biological parameters

To further characterize and better understand the intrinsic subtypes, we determined whether an association existed between intrinsic subtype and grade, node status, ER status, age, and tumor size in the combined test set. Two-way contingency table analysis showed significant association between grade and subtype, with HER2+/ER- and Basal-like tumors more likely to be grade 3 (Table 2). The Cramer's V statistic[28], which measures the strength of association between two variables in a contingency table, indicated a substantial association (Cramer's V > 0.36) between grade and subtype. Two-way contingency table analysis did not show significant association between node status and subtype (p = 0.44), but did show significant association between ER status and subtype (p < 0.0001; Cramer's V = 0.72) and between tumor size and subtype (p = 0.01; Cramer's V = 0.17). As would be expected, ER+ tumors were more likely to be LumA or LumB. As indicated by the low Cramer's V (Cramer's V < 0.19 indicates a low relationship), tumor size and subtype were not strongly correlated.

To determine association between age and subtype, we used an unpaired Student's t-test to compare the average ages of diagnosis of each tumor subtype. Interestingly, the average age of diagnosis for HER2+/ER- tumors was significantly less than that for all other tumor types. The average age of diagnosis for LumA tumors was significantly greater than that for LumB tumors.

### Derivation and application of a Single Sample Predictor

A caveat to the above analyses is that our classifications were based upon hierarchical clustering, which is a powerful tool for intrinsic class discovery, but which is not suited for individual sample classification because to classify a new sample would require a reanalysis of all samples. Therefore, we wanted to create an unchanging and objective method to classify tumors according to intrinsic subtype that could be clinically applicable. To this end, we developed a Single Sample Predictor (SSP) using the combined test set hierarchically clustered using the 306 Intrinsic/UNC genes (Figure 1). For the SSP, a mean expression profile (i.e. centroid) was created for each subtype that was significant in the multivariate analysis (LumA, LumB, Basal-like, HER2+/ER-) and for the Normal Breast-like group using the combined test set (Figure 2). Next, any new sample is then compared to each Centroid and assigned by the SSP to the nearest subtype/centroid as determined by Spearman correlation (note: this SSP is based on methods developed by Tibshirani and colleagues[3,29,30]); thus, the SSP contains five different idealized profiles, and any new sample is compared to each of the five profiles and assigned a profile label (i.e. subtype name) based upon the single idealized profile it most resembled.

To validate the SSP, we tested it on two additional datasets not used previously. The first was the 60-patient Ma et al. dataset, which represents a group of early stage ER+ tamoxifen-treated patients [6]. The SSP classified these samples as follows: 2 Basal-like, 2 HER2+/ER-, 12 Normal Breast-like, 34 LumA, and 9 LumB. The 2 Basal-like and 2 HER2+/ER- assigned samples were excluded from a survival analysis because they were too few for a meaningful survival analysis and possibly were misclassified ER-negative tumors. Among the remaining samples the SSP classification was a significant predictor of RFS (p = 0.04, Figure 3B), due to the poor outcome of the LumB group. Next, we applied the SSP to a 96-sample test set of local only (surgery)-treated patients from Chang et al. [31]. The tumor groups identified by the SSP showed significant differences in RFS (Figure 3C, p = 0.0006) and OS (p = 0.001, data not shown) in Kaplan-Meier analysis, with the poor outcome groups as expected: LumB, Basal-like, and HER2+/ER-. Thus, the SSP identified tumor groups that are truly prognostic and have significantly different outcomes as was seen before: namely, LumA always has the most favorable outcome, while LumB, Basal-like and HER2+/ER- do poorly[2,3,9,19].

We also applied the SSP onto the 105-sample dataset used to derive the Intrinsic/UNC gene list, which is technically not a test set for the SSP because it was used to derive the Intrinsic/UNC gene set. The tumor groups identified by the SSP showed significantly different RFS (Figure 3D, p = 0.02) and OS (p = 0.03, data not shown) in Kaplan-Meier analysis with the poor outcome groups again being LumB, Basal-like, and HER2+/ER-. A subset of the 105-sample dataset (48 in total) had been previously characterized using an immunohistochemical (IHC) analysis[32], which showed that (1) all 18 Basal-like tumors were ER-negative and HER2-negative (defined as not having a 3+ score on HER2 IHC analysis), (2) all 18 luminal subtype tumors were ER-positive and HER2-negative, and (3) all 12 HER2+/ER- subtype tumors were ER-negative and 11 out of these 12 showed HER2-overexpression (defined as having a 3+ score on HER2 IHC analysis). Thus, the SSP correlated with many standard clinical parameters, and was also able to identify clinically relevant groups (i.e. LumA vs. LumB) not identifiable using the standard clinical assays, thus indicating potential value as an objective classification method that should be developed further as a clinically applicable test.

## Discussion

The development and validation of gene sets for cancer patients requires significant resources because large training and test sets are required to achieve robust results. In fact, microarray studies are often criticized for a lack of rigorous validation due to small sample sizes [17,18]. Therefore, we utilized a previously described microarray data set combining method (Distance Weighted Discrimination) to create a large validation test set of over 300 tumors, and used it to validate a newly derived gene list for breast cancer prognostication and prediction. This approach allowed us to perform a multivariate analysis in which we show for the first time that the intrinsic subtype classification adds valuable information in the presence of five standard clinical parameters. We believe this combined test set is a valid test set for use in our analysis because after the multiple data sets were combined, the prognostic abilities of the standard clinical variables such as ER and grade remained intact.

The remarkable power of our DWD-based approach is indicated by the fact that although samples came from different platforms, hierarchical clustering analysis of the combined data set managed to group samples and genes based upon biology, and not some artifact caused by combining the data sets together. Evidence that this grouping reflected biology and not some artifact comes from (1) the finding that various Gene Ontology terms were significantly over-represented relative to chance in individual gene clusters seen in this analysis and (2) the groupings of the samples showed inter-dataset mixing and were significant predictors of outcome in univariate Kaplan-Meier and multivariate Cox analysis. It is also remarkable that this classification was successful in predicting outcome despite the fact that the Intrinsic/UNC gene set was reduced from 1300 genes to 306 genes in the combined test set; this indicates the robust nature of the intrinsic subtypes as defined by the new Intrinsic/UNC gene list.

One of the accomplishments of this manuscript was to develop an unchanging and objective intrinsic subtype predictor that could be used routinely in the clinical setting. This was accomplished by first identifying a new intrinsic gene set and then using this set to develop the Single Sample Predictor (SSP) that was shown here to be both prognostic on the local therapy-only patient subset from Chang et al. [31] and predictive of outcomes on the ER+ tamoxifen-treated data set of Ma et al. [6]. Many other gene expression based predictors for breast cancer patients have been developed, and in a complementary publication[33], we tested the intrinsic subtype SSP developed here, relative to those predictions made by four other previously published breast cancer prognostic/predictive gene sets using a single patient/tumor set of 295 cases; the four other expression-based predictors used were (1) the "70-gene" Good *vs*. Poor outcome predictor developed by van't Veer and colleagues[5,11], (2) the "Wound-Response" profile developed by Chang et al[31,34], (3) the "Recurrence Score (RS)" profile developed by Paik et al. [10], and (4) the 2-gene (HOXB13:IL17BR) ratio predictor developed by Ma et al. [6]. The results showed that of samples classified as Basal-like, HER2+/ER-, or LumB by the SSP, 93–100% were classified by the 70-gene, RS and Wound-Response predictors as being in each predictor's bad prognosis group. These data suggest that a high concordance exists across these multiple predictors, in particular the RS, 70-gene and Intrinsic Subtypes; thus, the new intrinsic gene list and classification method developed here, when compared to other predictors as accomplished in Fan et al. [33], showed that a high concordance across predictors exists, which provides additional validation for each predictor.

## Conclusion

The results of this study advance our current knowledge of the intrinsic breast tumor subtypes and provides an objective method (SSP) for prospectively classifying tumors that could be used in the clinical setting. More broadly speaking, our findings show that while the individual brushstrokes (*i.e*. genes) may sometimes show discordance across data sets, the portraits created by the combined patterns of the individual brushstrokes is conserved and recognizable across datasets because of the similarities to the family portrait [24]. Moreover, these data show that the breast tumor intrinsic subtypes identified using the Intrinsic/UNC gene list can be generalized to many different patient sets, both treated and untreated.

## Methods

### Sample collection, RNA isolation and microarray hybridization

105 fresh frozen breast tumor samples and 9 normal breast samples were obtained using IRB-approved protocols at 4 institutions: the University of North Carolina at Chapel Hill (UNC-CH), The University of Utah, Thomas Jefferson University, and the University of Chicago. This sample set represents an ethnically and geographically diverse cohort. Additional file 2 contains clinical data for these samples. Patients were heterogeneously treated according to the standard of care dictated by disease stage, ER and HER2 status.

Total RNA was purified from each sample using the Qiagen RNAeasy Kit. RNA integrity was determined using the RNA 6000 Nano LabChip Kit and Agilent 2100 Bioanalyzer. Total RNA amplification and labeling were done as previously described in [35]. Microarray hybridizations were performed using Agilent Human oligonucleotide (1Av1, 1Av2 and custom designed 1Av1-based) microarrays using 2 μg of Cy3-labeled common reference sample that is a modified version of the Stratagene Human Universal Reference[36], and 2 μg of Cy5-labeled experimental sample. Microarrays were hybridized overnight, washed, dried, and scanned as described in [35]. The image files were analyzed with GenePix Pro 4.1 and loaded into the UNC-CH Microarray Database[37] where a Lowess normalization procedure was performed to adjust the Cy3 and Cy5 channels[38]. All primary microarray data associated with this study are available at [39], 2006 #2192} and in the GEO[40] under the accession number of GSE1992, series GSM34424-GSM34568.

### Identification of the intrinsic gene set

We derived a new breast tumor intrinsic gene set, referred to as the "Intrinsic/UNC" list, using a training set composed of the 105 tumor samples described above, 9 normal breast samples, and 26 sample pairs (in total, represented by 146 microarrays). 15, 9, and 2 of the 26 sample pairs were different physical pieces of the same tumor (taken at the same time point), tumor-metastasis pairs and normal sample pairs, respectively. The background subtracted, Lowess normalized log_2 _ratio of Cy5 to Cy3 intensity values were first filtered to select genes that had a signal intensity of at least 30 units above background in both the Cy5 and Cy3 channels. Only genes that met these criteria in at least 70% of the 146 microarrays were included for subsequent analysis. Next, we performed an "intrinsic" analysis as described previously[3] using the 26 sample pairs and 86 additional microarrays. An intrinsic analysis identifies genes showing low variability in expression within paired samples but high variability in expression across different tumors; for each gene a ratio of "within-pair variance" to "between-subject variance" is computed. Genes with ratios below one standard deviation of the mean ratio were defined as "intrinsic". This analysis resulted in 1410 microarray elements representing 1300 genes being identified as "intrinsic". In order to obtain an estimate of the number of false-positive intrinsic genes, we permuted the sample labels to generate 26 random pairs and 86 non-paired samples. This permutation was performed 100 times and the intrinsic scores were calculated for each. These permuted scores were used to determine a threshold on the intrinsic score corresponding to a false discovery rate (FDR) less than 1%. The selected threshold resulted in 1410 microarray features being called significant with a median FDR = 0.3% and 90th percentile FDR = 0.5%. (See Tusher *et al. *for a complete description of this calculation [41]).

### Creation and analyses of the combined test set

The independent test set was a 315-sample "combined test set" consisting of three DNA microarray datasets (Sorlie *et al*. 2001 and 2003[2,3], van't Veer *et al*. 2002[5] and Sotiriou *et al. *2003[19]). To combine these datasets obtained from different microarray platforms, we performed the following pre-processing methods. First, the R/G ratios in each dataset were log_2 _transformed and Lowess normalized[38]. Next, missing values were k-NN imputed[42]. Gene annotations from each dataset were converted into UniGene Cluster IDs (UCIDs, Build 161) using the SOURCE database[43], and multiple occurrences of a UCID were collapsed by taking the median value for that ID within each experiment and platform, which resulted in ~2800 genes having expression data in all three datasets. Next, Distance Weighted Discrimination[20] was performed in a pair-wise fashion by first combining the Sorlie et al. and Sotiriou et al. datasets, and then combining this with the van't Veer et al. dataset to make a single dataset. In the final pre-processing step, each microarray experiment was normalized such that each column/experimental sample was standardized to N(0,1), and each row/gene was median centered. 306 of the 1300 Intrinsic/UNC genes had microarray data present in the combined test set and were used in a two-way average-linkage hierarchical cluster analysis [44]. Cluster results were visualized using the program "Treeview".

### Derivation of the Single Sample Predictor

The Single Sample Predictor (SSP) is a Nearest Centroid-based method based upon the work of Hastie and Tibshirani [3,45,46]. Our SSP classifies an individual sample according to its nearest centroid as determined by Spearman correlation. To derive our SSP, we utilized the 315-sample combined test set from Figure2 to create centroids for each of the five intrinsic subtypes (LumA, LumB, HER2+/ER-, Basal-like and Normal Breast-like). Please note that we did not create a centroid for the IFN group because it failed significance in multivariate testing, but did create a centroid for the Normal Breast-like group because we feel it is important to be able to identify true normal samples; an H&E examination of most tumor samples falling into the Normal Breast-like category shows that this is occurring mainly because of too much normal tissue contamination.

To create each intrinsic subtype centroid, we averaged the gene expression profiles for samples clearly assigned to each subtype (limiting the analysis to 249 of the 315 samples) using the hierarchical clustering dendrogram as a guide (Figure 2). We then applied the SSP to two independent test datasets: (1) the Ma et al. 60-sample ER+ tamoxifen-treated tumor dataset and (2) the Chang et al. 96-sample local only-treated tumor dataset. By matching UCIDs, microarray data for as many as possible of the 306 Intrinsic/UNC genes was obtained from these 2 datasets. To remove microarray platform/source systematic biases, we applied DWD to the 2 test datasets relative to the combined test set. The SSP was then used to classify tumors by intrinsic subtype in these 2 test datasets. Using similar methods, the SSP was also applied to the 105-sample training set used to derive the intrinsic/UNC gene set.

### Survival analyses

Kaplan-Meier survival plots were compared using the Cox-Mantel log-rank test in WinSTAT for Excel (R. Fitch Software). Two-way contingency table analysis and unpaired Student's t-test were done using WinSTAT. For the "combined test set", multivariate Cox proportional hazards analysis was performed using SAS (Cary, NC).

## Authors' contributions

C.M.P. was the Principal Investigator and instigated the study, helped with design and wrote the paper, while J.P., O.I.O, and P.S.B. were the Principal Investigators at each of the three other participating institution and were involved in the study design, implementation and manuscript writing. C.F., J.S.M., B.F.Q., A.N., and J.P. were responsible for the statistical analyses and some writing. Z.H and X.H. performed all of the tumor RNA preparation and microarray experiments and were involved in the writing. J.W. and Y.L. were responsible for all data management and some data analysis. C.L. was responsible for the pathological assessment of most tumor samples and was involved in the writing. M.E. and D.O. were involved in data analysis, interpretation and writing. Tumor sample collection, clinical data acquisition and interpretation was accomplished by L.C., M.E., R.N., M.T., A.R.O., D.D., L.P., E.N., M.M., H.H., M.M., J.F.Q., L.R.S., E.R., and L.D., and it should be noted that this was separately accomplished at four institutions.

## Supplementary Material

Additional File 2 Supplemental Table 1. Clinical and microarray information associated with each patient in the 105-sample training dataset. Click here for file

Additional File 1 Supplemental Figure 1. Complete hierarchical cluster diagram of the 315-sample combined test set analyzed using the Intrinsic/UNC gene set, which was reduced to 306 genes based upon the gene overlap between datasets. Sorlie *et al. *sample names begin with the letters "BC", Sotiriou *et al. *sample names begin with "Exp", and van't Veer *et al. *sample names begin with "sample". Click here for file

## Figures and Tables

**Table 1:** Multivariate Cox proportional hazards analysis of (A) standard clinical factors alone, or with (B) the Intrinsic Subtypes in relation to Relapse-Free Survival for the 315-sample combined test set. Size was a binary variable (0 = diameter of 2 cm or less, 1 = greater than 2 cm); node status was a binary variable (0 = no positive nodes, 1 = one or more positive nodes); age was a continuous variable formatted as decade-years. Hazard ratios for Intrinsic Subtypes were calculated relative to the Luminal A subtype. Variables found to be significant (p < 0.05) in the Cox proportional hazards model are shown in bold.

**A.**	**Relapse-Free survival**	
**Variable**	**Hazard Ratio (95% CI)**	**p-value**

Age, per decade	1.04 (0.90–1.20)	0.64
ER status	**0.59 **(0.41–0.83)	**0.003**
Node status	1.41 (0.98–2.04)	0.07
Tumor grade 2 *vs*. 1	**2.41 **(1.08–5.36)	**0.032**
Tumor grade 3 *vs*. 1	**3.98 **(1.80–8.82)	**0.0007**
Size	**1.60 **(1.31–1.95)	**<0.0001**

**B.**	**Relapse-Free survival**	

**Variable**	**Hazard Ratio (95% CI)**	**p-value**

Age, per decade	1.08 (0.94–1.24)	0.29
ER status	0.69 (0.42–1.13)	0.14
Node status	1.35 (0.92–1.98)	0.13
Tumor grade 2 *vs*. 1	1.88 (0.82–4.32)	0.14
Tumor grade 3 *vs*. 1	**2.58 **(1.08–6.12)	**0.03**
Size	**1.59 **(1.30–1.95)	**<0.0001**
Basal-like vs. LumA	**2.02 **(1.05–3.90)	**0.036**
HER2+/ER- vs. LumA	**3.47 **(1.78–6.76)	**0.0003**
LumB vs. LumA	**1.92 **(1.07–3.45)	**0.028**
IFN vs. LumA	1.40 (0.67–2.91)	0.37
Normal-like vs. LumA	1.56 (0.59–4.16)	0.37

**Table 2:** Association between tumor histologic grade and intrinsic subtype in the 315-sample combined test set.

	**Intrinsic Subtype**				
**Two-way contingency table**	**LumA ** (#of pts.)	**LumB ** (#of pts.)	**IFN ** (#of pts.)	**HER2+/ER- ** (#of pts.)	**Basal-like ** (#of pts.)

**Grade**					
1 (well)	29	2	1	0	1
2 (intermediate)	45	26	8	6	16
3 (poor)	15	32	16	21	67
**Statistics for two-way contingency table analysis**					
p-value† <0.0001					
Cramer's V†† 0.42					

†p-value calculated from Chi-square test on contingency table. ††Cramer's V statistic (value can range from 0 to 1) measures the strength of association between the two variables analyzed in the contingency table, with 1 indicating perfect association and 0 indicating no association.
