# Supplementary material for: The molecular portraits of breast tumors are conserved across microarray platforms
Source: BMC Genomics. 2006 Apr 27;7:96. doi: 10.1186/1471-2164-7-96 (PMC1468408; doi:10.1186/1471-2164-7-96)
Supplement: Additional File 2 — Supplemental Table 1. Clinical and microarray information associated with each patient in the 105-sample training dataset. [file 1471-2164-7-96-S2.pdf]

GEO sample names	intrinsic pair or RNA replicate	training or test set sample	Age	ER (1=positive; 0=negative)	Node status (1=positive; 0=negative)	Grade	Size (1= <=2cm; 2= >2cm to <=5cm; 3=>5cm; 4=any size with direct extension	RFS event (0=no relapse, 1=relapsed or died of disease)	RFS months	Overall Survival Event (0=alive, 1=DOD or DOC)	Overall suvival months	Array Name	order in Figure 2 cluster	1 sample/array per patient	Spearman SSP as Figure 2	Intrinsic Subtype According to Figure 2	Target Source 2 (Cy5)	Target source 1 (Cy3)
020573-BC-Primary	pair 1	training	41	1	1	3	3	1	10	0	22	H1A9529-0087C	9	1	HER2+/ER-	HER2+/ER	Breast primary for a patient with an associated brain metastases (	Stratagene Human Universal Reference that contained 1/10 added MCF7 and ME16C RNAs
020577-BC-Brain-Met(=7	pair 1	training										H1A5949-019	8		HER2+/ER-	HER2+/ER	Brain metastases with an associated primary 020573	Stratagene Human Universal Reference that contained 1/10 added MCF7 and ME16C RNAs
030064-BC-Brain-Met		training	32	1	1	2	1	1	51	0	64	H1A5365-900	4	2	HER2+/ER-	HER2+/ER	Brain metastases from breast	Stratagene Human Universal Reference that contained 1/10 added MCF7 and ME16C RNAs
030432-BC-Brain-Met	pair 2	training										H1A9529-042C	123	3	LuminalB	Luminal	Brain metastases from breast	Stratagene Human Universal Reference that contained 1/10 added MCF7 and ME16C RNAs
030432-BC-Brain-Met-Int	pair 2	training										H1A9529-0001C	122		LuminalB	Luminal	Brain metastases from breast	Stratagene Human Universal Reference that contained 1/10 added MCF7 and ME16C RNAs
030521-BC-Brain-Met	pair 3	training										H1A7411-0109-C	6	4	HER2+/ER-	HER2+/ER	Brain metastases from breast	Stratagene Human Universal Reference that contained 1/10 added MCF7 and ME16C RNAs
030521-BC-Brain-Met-Int	pair 3	training										H1A7411-0110-C	5		HER2+/ER-	HER2+/ER	Brain metastases from breast	Stratagene Human Universal Reference that contained 1/10 added MCF7 and ME16C RNAs
A1-01-Right-Lung-VIM	pair 4	training										H1A6261-1489V2_Na	31	5	Basal	Basal	Autopsy patient lung metastases from breast	Stratagene Human Universal Reference that contained 1/10 added MCF7 and ME16C RNAs
A1-17-Left-Primary-VIM	pair 4	training	64	0	1	3	4	1	2	1	2	H1A9529-0094C	30		Basal	Basal	Autopsy patient breast primary	Stratagene Human Universal Reference that contained 1/10 added MCF7 and ME16C RNAs
A4-LLL-Lung-Met	pair 5	training	44	1	1	3	4	1	22	1	22	H1A6912-6852V2	23	6	Basal	HER2+/ER	Autopsy patient lung metastases from breast	Stratagene Human Universal Reference that contained 1/10 added MCF7 and ME16C RNAs
A4-LUL-Lung-Met	pair 5	training										H1A6912-6851V2	22		Basal	HER2+/ER	Autopsy patient lung metastases from breast	Stratagene Human Universal Reference that contained 1/10 added MCF7 and ME16C RNAs
A5-Skin#1-Right-Met	pair 6	training	65	0	1	3	4	1	26	1	26	H1A6912-6853V2	56	7	Basal	Basal	Autopsy patient skin metastases from breast	Stratagene Human Universal Reference that contained 1/10 added MCF7 and ME16C RNAs
A5-Skin-Left-Met	pair 6	training										H1A6912-6955V2	55		Basal	Basal	Autopsy patient skin metastases from breast	Stratagene Human Universal Reference that contained 1/10 added MCF7 and ME16C RNAs
BC00004	replicate pair,	training	67	0	0	2	1	0	118	0	118	H1A9529-30C_Na	99	8	LuminalA	Luminal	Breast Tumor	Stratagene Human Universal Reference that contained 1/10 added MCF7 and ME16C RNAs
BC00004-Repeat1	replicate pair,	training										H1A6433-2600V2	100		LuminalA	Luminal	Breast Tumor	Stratagene Human Universal Reference that contained 1/10 added MCF7 and ME16C RNAs
BC00010	replicate pair,	training	47	0	1	2	3	1	16	1	16	H1A9529-39C_Na	12	9	HER2+/ER-	HER2+/ER	Breast Tumor	Stratagene Human Universal Reference that contained 1/10 added MCF7 and ME16C RNAs
BC00010-Repeat1	replicate pair,	training										H1A6433-2601V2	13		HER2+/ER-	HER2+/ER	Breast Tumor	Stratagene Human Universal Reference that contained 1/10 added MCF7 and ME16C RNAs
BC00014	replicate pair,	training	69	0	1	3	4	1	18	1	23	H1A9529-0045Na_C	142	10	LuminalA	NB	Breast Tumor	Stratagene Human Universal Reference that contained 1/10 added MCF7 and ME16C RNAs
BC00014-Repeat1	replicate pair,	training										H1A6730-644	143		HER2+/ER-	NB	Breast Tumor	Stratagene Human Universal Reference that contained 1/10 added MCF7 and ME16C RNAs
BC00024T		training	68	0	1	3	3	1	3	1	3	H1A_C_0077	35	11	Basal	Basal	Breast Tumor	Stratagene Human Universal Reference that contained 1/10 added MCF7 and ME16C RNAs
BC00029T		training	44	0	1	3		0	62	0	62	H1A_C_0078	26	12	Basal	Basal	Breast Tumor	Stratagene Human Universal Reference that contained 1/10 added MCF7 and ME16C RNAs
BC00034		training	68	0	0	2	1	0	81	0	81	H1A9529-0041C_Na	121	13	LuminalA	Luminal	Breast Tumor	Stratagene Human Universal Reference that contained 1/10 added MCF7 and ME16C RNAs
BC00036-Repeat1	replicate pair,	training										H1A6261-2980V2_Na	19	14	HER2+/ER-	HER2+/ER	Breast Tumor	Stratagene Human Universal Reference that contained 1/10 added MCF7 and ME16C RNAs
BC00036T	replicate pair,	training	55	0	1	2	2	0	10	0	10	H1A_C_0079	18		HER2+/ER-	HER2+/ER	Breast Tumor	Stratagene Human Universal Reference that contained 1/10 added MCF7 and ME16C RNAs
BC00041		training	46	0	0	3	2	1	13	1	29	H1A9529-039C	45	15	Basal	Basal	Breast Tumor	Stratagene Human Universal Reference that contained 1/10 added MCF7 and ME16C RNAs
BC00043T		training	43	0	0	3	2	0	76	0	76	H1A_C_0080	42	16	Basal	Basal	Breast Tumor	Stratagene Human Universal Reference that contained 1/10 added MCF7 and ME16C RNAs
BC00049T		training	43	0	1	3	2	1	48	0	72	H1A_C_0081	41	17	Basal	Basal	Breast Tumor	Stratagene Human Universal Reference that contained 1/10 added MCF7 and ME16C RNAs
BC00051		training	51	0	0	2	2	0	68	0	68	H1A9529-0040C_Na	21	18	HER2+/ER-	HER2+/ER	Breast Tumor	Stratagene Human Universal Reference that contained 1/10 added MCF7 and ME16C RNAs
BC00052-post-chemo	pair 7	training	47	0	1	3	2	1	14	1	18	H1A9529-040C	3	19	HER2+/ER-	HER2+/ER	Breast primary post chemotherapy	Stratagene Human Universal Reference that contained 1/10 added MCF7 and ME16C RNAs
BC00052-post-chemo-Int	pair 7	training										H1A6261-2607V2	2		HER2+/ER-	HER2+/ER	Breast primary post chemotherapy	Stratagene Human Universal Reference that contained 1/10 added MCF7 and ME16C RNAs
BC00053		training	71	0	1	3	2	1	27	1	28	H1A6433-2608V2	16	20	NB	HER2+/ER	Breast Tumor	Stratagene Human Universal Reference that contained 1/10 added MCF7 and ME16C RNAs
BC00057-post-chemo		training	51	0	1	3	4	1	8	1	12	H1A6260-731	69	21	HER2+/ER-	Unclass	Breast primary post chemotherapy	Stratagene Human Universal Reference that contained 1/10 added MCF7 and ME16C RNAs
BC00064-RECUR		training	44	0		3	1	1	10	1	47	H1A6357_3552	36	22	Basal	Basal	Breast tumor recurrence	Stratagene Human Universal Reference that contained 1/10 added MCF7 and ME16C RNAs
BC00066		training	43	0	1	3	3	1	18	1	18	H1A9529-022-C	145	23	LuminalA	Unclass	Breast Tumor	Stratagene Human Universal Reference that contained 1/10 added MCF7 and ME16C RNAs
BC00070	pair 8	training	38	0	0	2	2	1	22	1	25	H1A9529-0096C	40	24	Basal	Basal	Breast Tumor	Stratagene Human Universal Reference that contained 1/10 added MCF7 and ME16C RNAs
BC00070-Int	pair 8	training										H1A6433-2610V2	39		Basal	Basal	Breast Tumor	Stratagene Human Universal Reference that contained 1/10 added MCF7 and ME16C RNAs
BC00071		training	33	0	1	2	2	1	16	1	47	H1A9529-0089C	24	25	HER2+/ER-	HER2+/ER	Breast Tumor	Stratagene Human Universal Reference that contained 1/10 added MCF7 and ME16C RNAs
BC00078T		training	68	0	1	3	3	0	12	1	12	H1A4110_3579	38	26	Basal	Basal	Breast Tumor	Stratagene Human Universal Reference that contained 1/10 added MCF7 and ME16C RNAs
BC00082T		training	84	0	0	3	2	0	27	1	27	H1A4110_3580	37	27	Basal	Basal	Breast Tumor	Stratagene Human Universal Reference that contained 1/10 added MCF7 and ME16C RNAs
BC00085		training	24	0	0	2	1	0	19	0	19	H1A6260-732	52	28	Basal	Basal	Breast Tumor	Stratagene Human Universal Reference that contained 1/10 added MCF7 and ME16C RNAs
BR00-0284	pair 9	training	63	0	0	3	3	0	43	0	43	H1A6433-2611V2	11	29	HER2+/ER-	HER2+/ER	Breast Tumor	Stratagene Human Universal Reference that contained 1/10 added MCF7 and ME16C RNAs
BR00-0284-Int	pair 9	training										H1A6433-2987V2	10		HER2+/ER-	HER2+/ER	Breast Tumor	Stratagene Human Universal Reference that contained 1/10 added MCF7 and ME16C RNAs
BR00-0344	replicate pair,	training	65	1	1	3	2	1	7	1	30	H1A9529-028C_Na	72	30	LuminalB	Luminal	Breast Tumor	Stratagene Human Universal Reference that contained 1/10 added MCF7 and ME16C RNAs
BR00-0344-Repeat1	replicate pair,	training										H1A6433-2598V2	73		LuminalB	Luminal	Breast Tumor	Stratagene Human Universal Reference that contained 1/10 added MCF7 and ME16C RNAs
BR00-0365		training	43	1	1	3	4	0	22	0	22	H1A_013Na_C	124	31	LuminalB	Luminal	Breast Tumor	Stratagene Human Universal Reference that contained 1/10 added MCF7 and ME16C RNAs
BR00-0387	replicate pair,	training	57	1	1	2	4	1	6	0	51	H1A9529-025C_Na	102	32	LuminalA	Luminal	Breast Tumor	Stratagene Human Universal Reference that contained 1/10 added MCF7 and ME16C RNAs
BR00-0387-Repeat1	replicate pair,	training										H1A6433-2597V2	103		LuminalA	Luminal	Breast Tumor	Stratagene Human Universal Reference that contained 1/10 added MCF7 and ME16C RNAs
BR00-0504		training	88	1	1	1	2	0	39	0	39	H1A6433-2988V2	94	33	LuminalA	Luminal	Breast Tumor	Stratagene Human Universal Reference that contained 1/10 added MCF7 and ME16C RNAs
BR00-0572-VIM	pair 10	training	45	0	1	3	3	1	11	0	42	H1A6433-2599V2	57	34	Basal	Basal	Breast Tumor	Stratagene Human Universal Reference that contained 1/10 added MCF7 and ME16C RNAs
BR00-0572-VIM-Int	pair 10	training										H1A6433-2989V2	58		Basal	Basal	Breast Tumor	Stratagene Human Universal Reference that contained 1/10 added MCF7 and ME16C RNAs
BR00-0587-GATA3		training	68	1	0	3	2	0	37	0	37	H1A6433-2990V2	93	35	LuminalA	Luminal	Breast Tumor	Stratagene Human Universal Reference that contained 1/10 added MCF7 and ME16C RNAs
BR01-0123		training	49	0	1	2	3	1	31	0	41	H1A9529-0092C	20	36	HER2+/ER-	HER2+/ER	Breast Tumor	Stratagene Human Universal Reference that contained 1/10 added MCF7 and ME16C RNAs
BR01-0125		training	40	1	1	2	3	0	33	0	33	H1A6433-2992V2	79	37	LuminalB	Luminal	Breast Tumor	Stratagene Human Universal Reference that contained 1/10 added MCF7 and ME16C RNAs
BR01-0246		training	36	1	1	2	2	0	31	0	31	H1A9529-0093C	76	38	LuminalA	Luminal	Breast Tumor	Stratagene Human Universal Reference that contained 1/10 added MCF7 and ME16C RNAs
BR01-0349		training	37	1	1	3	3	1	3	1	24	H1A9529-0047C	128	39	LuminalB	Luminal	Breast Tumor	Stratagene Human Universal Reference that contained 1/10 added MCF7 and ME16C RNAs
BR94-1083		training	48	0	1	3	1	1	23	1	47	H1A6912-6957V2	146	40	LuminalA	Unclass	Breast Tumor	Stratagene Human Universal Reference that contained 1/10 added MCF7 and ME16C RNAs
BR95-0017		training	52	1	1	3	2	1	36	1	64	H1A6261-1488V2	127	41	LuminalB	Luminal	Breast Tumor	Stratagene Human Universal Reference that contained 1/10 added MCF7 and ME16C RNAs
BR95-0035		training	74	0	1	3	2	0	106	0	106	H1A6912-6984V2	92	42	LuminalA	Luminal	Breast Tumor	Stratagene Human Universal Reference that contained 1/10 added MCF7 and ME16C RNAs
BR95-0152		training	72	1	0	3	4	1	26	0	101	H1A6912-6985V2	25	43	HER2+/ER-	HER2+/ER	Breast Tumor	Stratagene Human Universal Reference that contained 1/10 added MCF7 and ME16C RNAs
BR95-0184		training	74	1	0	3	2	0	96	0	96	H1A6912-6986V2	104	44	LuminalB	Luminal	Breast Tumor	Stratagene Human Universal Reference that contained 1/10 added MCF7 and ME16C RNAs
BR96-0014		training	47	1	1	1	4	0	96	0	96	H1A6912-6987V2	95	45	LuminalA	Luminal	Breast Tumor	Stratagene Human Universal Reference that contained 1/10 added MCF7 and ME16C RNAs
BR97-0137-VIM	pair 11	training										H1A6261-1489V2	33	46	Basal	Basal	Breast Tumor	Stratagene Human Universal Reference that contained 1/10 added MCF7 and ME16C RNAs
BR97-0137-VIM-Int	pair 11	training	55	0	1	3	3	1	20	1	21	H1A6261-1490V2	34		Basal	Basal	Breast Tumor	Stratagene Human Universal Reference that contained 1/10 added MCF7 and ME16C RNAs
BR97-1045		training	44	1	1	2	2	0	31	0	31	H1A9529-0048C	120	47	LuminalA	Luminal	Breast Tumor	Stratagene Human Universal Reference that contained 1/10 added MCF7 and ME16C RNAs
BR98-0161		training	57	0	1	2	2	1	36	1	60	H1A9529-0049C	105	48	LuminalB	Luminal	Breast Tumor	Stratagene Human Universal Reference that contained 1/10 added MCF7 and ME16C RNAs
BR98-0261	replicate pair,	training	44	0	0	3	2	0	65	0	65	H1A014Na_C	64	49	Basal	Basal	Breast Tumor	Stratagene Human Universal Reference that contained 1/10 added MCF7 and ME16C RNAs

BR98-0261-Repeat1	replicate pair,	training										H1A9529-0050C	63		Basal	Basal	Breast Tumor	Stratagene Human Universal Reference that contained 1/10 added MCF7 and ME16C RNAs
BR99-0207-GATA3	pair 12	training										H1A7411-0066-C	113	50	LuminalA	Luminal	Breast Tumor	Stratagene Human Universal Reference that contained 1/10 added MCF7 and ME16C RNAs
BR99-0207-GATA3-Int	pair 12	training	84	1	1	2	2	0	57	0	57	H1A012Na_C	114		LuminalA	Luminal	Breast Tumor	Stratagene Human Universal Reference that contained 1/10 added MCF7 and ME16C RNAs
BR99-0348-GATA3	pair 13	training	85	1	0	2	2	0	32	0	32	H1A9529-0051C	98	51	LuminalA	Luminal	Breast Tumor	Stratagene Human Universal Reference that contained 1/10 added MCF7 and ME16C RNAs
BR99-0348-GATA3-Int	pair 13	training										H1A7411-0082-C	97		LuminalA	Luminal	Breast Tumor	Stratagene Human Universal Reference that contained 1/10 added MCF7 and ME16C RNAs
Normal-Breast-1		training										H1A6261-2992V2_Na	131		NB	NB	Normal breast tissue from BD	Stratagene Human Universal Reference that contained 1/10 added MCF7 and ME16C RNAs
Normal-Breast-2	pair 25	training										H1A9529-0052C	139		NB	NB	Normal breast tissue from an individual	Stratagene Human Universal Reference that contained 1/10 added MCF7 and ME16C RNAs
Normal-Breast-3		training										H1A9529-0095C	134		NB	NB	Normal breast tissue from an individual	Stratagene Human Universal Reference that contained 1/10 added MCF7 and ME16C RNAs
Normal-Breast-4	pair 26	training										H1A6261-2138V2_Na	136		NB	NB	Normal breast tissue from an individual	Stratagene Human Universal Reference that contained 1/10 added MCF7 and ME16C RNAs
Normal-Breast-5		training										H1A6261-2139V2_Na	140		NB	NB	Normal breast tissue from an individual	Stratagene Human Universal Reference that contained 1/10 added MCF7 and ME16C RNAs
Normal-Breast-6		training										H1A6261-2140V2_Na	133		NB	NB	Normal breast tissue from an individual	Stratagene Human Universal Reference that contained 1/10 added MCF7 and ME16C RNAs
Normal-Breast-7	pair 25	training										H1A9529-0053C	138		NB	NB	Normal breast tissue from an individual	Stratagene Human Universal Reference that contained 1/10 added MCF7 and ME16C RNAs
Normal-Breast-8		training										H1A6149-701S02	141		NB	NB	Normal breast tissue from an individual	Stratagene Human Universal Reference that contained 1/10 added MCF7 and ME16C RNAs
Normal-Breast-9	pair 26	training										H1A6261-1499V2_Na	135		NB	NB	Normal breast tissue from Strategene	Stratagene Human Universal Reference that contained 1/10 added MCF7 and ME16C RNAs
PB120-LN-Met		training	61	0	1	3	2	1	1	1	13	H1A6261-2597V2_Na	49	52	Basal	Basal	Lymph node metastasis	Stratagene Human Universal Reference that contained 1/10 added MCF7 and ME16C RNAs
PB126	pair 14	training	29	0	1	3	4	1	1	1	16	H1A6433-2761_V2	51	53	Basal	Basal	Breast primary with an associated lymph node metastasis	Stratagene Human Universal Reference that contained 1/10 added MCF7 and ME16C RNAs
PB126-LN-Met	pair 14	training										H1A6261-0922V2	50		Basal	Basal	Lymph node metastasis	Stratagene Human Universal Reference that contained 1/10 added MCF7 and ME16C RNAs
PB138	pair 15	training	58	0	0	2	2	0	30	0	30	H1A6261-0924V2	62	54	Basal	Basal	Breast Tumor	Stratagene Human Universal Reference that contained 1/10 added MCF7 and ME16C RNAs
PB138-Int	pair 15	training										H1A6261-0925V2	61		Basal	Basal	Breast Tumor	Stratagene Human Universal Reference that contained 1/10 added MCF7 and ME16C RNAs
PB149		training	41	1	0	1	2	0	34	0	34	H1A7411-0083-C	137	55	NB	NB	Breast Tumor	Stratagene Human Universal Reference that contained 1/10 added MCF7 and ME16C RNAs
PB158		training	86	1	0	3	3	0	30	0	30	H1A9529-0003C	71	56	LuminalB	Luminal	Breast Tumor	Stratagene Human Universal Reference that contained 1/10 added MCF7 and ME16C RNAs
PB184		training	50	1	0	3	1	0	29	0	29	H1A7411-0084-C	44	57	Basal	Basal	Breast Tumor	Stratagene Human Universal Reference that contained 1/10 added MCF7 and ME16C RNAs
PB196	pair 16	training	34	0	1	3	3	1	6	0	12	H1A6261-1497V2	27	58	Basal	Basal	Breast primary with an associated lymph node metastasis	Stratagene Human Universal Reference that contained 1/10 added MCF7 and ME16C RNAs
PB196-LN-Met	pair 16	training										H1A6261-0926V2	28		Basal	Basal	Lymph node metastasis	Stratagene Human Universal Reference that contained 1/10 added MCF7 and ME16C RNAs
PB205		training	39	0	1	2	4	0	5	0	5	H1A6433-2610V2_Na	68	59	Basal	Unclass	Breast Tumor	Stratagene Human Universal Reference that contained 1/10 added MCF7 and ME16C RNAs
PB249		training	36	1	1	3	1	0	8	0	8	H1A9529-0055C	110	60	LuminalB	Luminal	Breast Tumor	Stratagene Human Universal Reference that contained 1/10 added MCF7 and ME16C RNAs
PB255		training	56	1	1	3	2	0	4	0	4	H1A9529-0056C	96	61	LuminalA	Luminal	Breast Tumor	Stratagene Human Universal Reference that contained 1/10 added MCF7 and ME16C RNAs
PB258	pair 17	training	84	1	1	2	2					H1A9529-0057C	125	62	LuminalA	Luminal	Breast primary with an associated lymph node metastasis	Stratagene Human Universal Reference that contained 1/10 added MCF7 and ME16C RNAs
PB258-LN-Met	pair 17	training										H1A7411-0117-C	126		LuminalA	Luminal	Lymph node metastasis	Stratagene Human Universal Reference that contained 1/10 added MCF7 and ME16C RNAs
PB267		training	44	0	1	3	2	0	20	0	20	H1A7411-0085-C	43	63	Basal	Basal	Breast Tumor	Stratagene Human Universal Reference that contained 1/10 added MCF7 and ME16C RNAs
PB271		training	45	1	1	3	2	0	14	0	14	H1A9529-0058C	144	64	NB	NB	Breast Tumor	Stratagene Human Universal Reference that contained 1/10 added MCF7 and ME16C RNAs
PB277		training	46	1	1	3	2	0	12	0	12	H1A9529-0059C	75	65	LuminalB	Luminal	Breast Tumor	Stratagene Human Universal Reference that contained 1/10 added MCF7 and ME16C RNAs
PB293		training	56	1	0	2	2	0	11	0	11	H1A9529-0060C	81	66	LuminalB	Luminal	Breast Tumor	Stratagene Human Universal Reference that contained 1/10 added MCF7 and ME16C RNAs
PB297	pair 18	training	55	0	0	3	2	0	18	0	18	H1A9529-0061C	54	67	Basal	Basal	Breast Tumor	Stratagene Human Universal Reference that contained 1/10 added MCF7 and ME16C RNAs
PB297-Int	pair 18	training										H1A7411-0112-C	53		Basal	Basal	Breast Tumor	Stratagene Human Universal Reference that contained 1/10 added MCF7 and ME16C RNAs
PB307		training	35	1	0	3	3	0	9	0	9	H1A9529-0062C	109	68	LuminalB	Luminal	Breast Tumor	Stratagene Human Universal Reference that contained 1/10 added MCF7 and ME16C RNAs
PB311		training	48	1	1	3	3	0	14	0	14	H1A9529-0044C	70	69	LuminalB	Unclass	Breast Tumor	Stratagene Human Universal Reference that contained 1/10 added MCF7 and ME16C RNAs
PB314		training	51	0	1	3	3	0	21	0	21	H1A9529-0045C	7	70	HER2+/ER-	HER2+/ER	Breast Tumor	Stratagene Human Universal Reference that contained 1/10 added MCF7 and ME16C RNAs
PB334		training	50	0	0	3	1	0	19	0	19	H1A6433-2608V2_Na	48	71	Basal	Basal	Breast Tumor	Stratagene Human Universal Reference that contained 1/10 added MCF7 and ME16C RNAs
PB362		training	64	1	0	3	2	0	16	0	16	H1A6149-098	130	72	NB	NB	Breast Tumor	Stratagene Human Universal Reference that contained 1/10 added MCF7 and ME16C RNAs
PB370		training	67	1	1	3	2	0	20	0	20	H1A9529-0063C	101	73	LuminalA	Luminal	Breast Tumor	Stratagene Human Universal Reference that contained 1/10 added MCF7 and ME16C RNAs
PB376		training	50	0	0	3	2	0	15	0	15	H1A7411-0114-C	17	74	Basal	HER2+/ER	Breast Tumor	Stratagene Human Universal Reference that contained 1/10 added MCF7 and ME16C RNAs
PB377		training	77	1	0	3	2	0	18	0	18	H1A6989-8214-V2	29	75	Basal	Basal	Breast Tumor	Stratagene Human Universal Reference that contained 1/10 added MCF7 and ME16C RNAs
PB379		training	55	1	1	3	2	0	17	0	17	H1A6989-8215-V2	118	76	LuminalB	Luminal	Breast Tumor	Stratagene Human Universal Reference that contained 1/10 added MCF7 and ME16C RNAs
PB388		training	80	1	0	3	2	0	16	0	16	H1A6433-2809_V2	119	77	LuminalA	Luminal	Breast Tumor	Stratagene Human Universal Reference that contained 1/10 added MCF7 and ME16C RNAs
PB412	pair 19	training	58	0	0	3	2					H1A6433-2810_V2	60	78	Basal	Basal	Breast Tumor	Stratagene Human Universal Reference that contained 1/10 added MCF7 and ME16C RNAs
PB412-Int	pair 19	training										H1A6433-2811_V2	59		Basal	Basal	Breast Tumor	Stratagene Human Universal Reference that contained 1/10 added MCF7 and ME16C RNAs
PB413		training	63	1	1	2	2	0	9	0	9	H1A6989-8226-V2	115	79	LuminalA	Luminal	Breast Tumor	Stratagene Human Universal Reference that contained 1/10 added MCF7 and ME16C RNAs
PB419	pair 20	training	49	0	0	3	2	0	10	0	10	H1A6261-2140V2	66	80	Basal	Basal	Breast Tumor	Stratagene Human Universal Reference that contained 1/10 added MCF7 and ME16C RNAs
PB419-Int	pair 20	training										H1A6261-2141V2	65		Basal	Basal	Breast Tumor	Stratagene Human Universal Reference that contained 1/10 added MCF7 and ME16C RNAs
PB441		training	83	1	0	2	1	0	9	0	9	H1A7411-0113-C	90	81	LuminalA	Luminal	Breast Tumor	Stratagene Human Universal Reference that contained 1/10 added MCF7 and ME16C RNAs
PB455		training	52	0	1	2	3	0	9	0	9	H1A6433-2812_V2	14	82	HER2+/ER-	HER2+/ER	Breast Tumor	Stratagene Human Universal Reference that contained 1/10 added MCF7 and ME16C RNAs
PB475		training	50	1	0	2	2	0	2	0	2	H1A7411-0116-C	111	83	LuminalA	Luminal	Breast Tumor	Stratagene Human Universal Reference that contained 1/10 added MCF7 and ME16C RNAs
UB21		training	77	1	0	1	1	0	30	0	30	H1A6433-3007_V2	88	84	LuminalA	Luminal	Breast Tumor	Stratagene Human Universal Reference that contained 1/10 added MCF7 and ME16C RNAs
UB29		training	59	0	0	3	2	0	25	0	25	H1A6433-3010_V2	32	85	Basal	Basal	Breast Tumor	Stratagene Human Universal Reference that contained 1/10 added MCF7 and ME16C RNAs
UB37		training	42	0	1	3	1	0	25	0	25	H1A9529-0064C	15	86	HER2+/ER-	HER2+/ER	Breast Tumor	Stratagene Human Universal Reference that contained 1/10 added MCF7 and ME16C RNAs
UB38		training	50	1	0	1	1	0	20	0	20	H1A7411-0118-C	89	87	LuminalA	Luminal	Breast Tumor	Stratagene Human Universal Reference that contained 1/10 added MCF7 and ME16C RNAs
UB39		training	48	1	0	2	1	0	25	0	25	H1A6433-3011_V2	108	88	LuminalA	Luminal	Breast Tumor	Stratagene Human Universal Reference that contained 1/10 added MCF7 and ME16C RNAs
UB43		training	48	1	1	3	1	0	19	0	19	H1A6433-2272V2	74	89	LuminalA	Luminal	Breast Tumor	Stratagene Human Universal Reference that contained 1/10 added MCF7 and ME16C RNAs
UB44		training	50	1	1	2	2	0	24	0	24	H1A6433-2273_V2	86	90	LuminalA	Luminal	Breast Tumor	Stratagene Human Universal Reference that contained 1/10 added MCF7 and ME16C RNAs
UB45		training	46	1	1	2	2	0	21	0	21	H1A6433-2274_V2	85	91	LuminalA	Luminal	Breast Tumor	Stratagene Human Universal Reference that contained 1/10 added MCF7 and ME16C RNAs
UB55	pair 21	training	58	1	0	1	1	0	22	0	22	H1A7411-0120-C	84	92	LuminalA	Luminal	Breast Tumor	Stratagene Human Universal Reference that contained 1/10 added MCF7 and ME16C RNAs
UB55-Int	pair 21	training										H1A7411-0121-C	83		LuminalA	Luminal	Breast Tumor	Stratagene Human Universal Reference that contained 1/10 added MCF7 and ME16C RNAs
UB56		training	63	1	1	2	1	0	9	0	9	H1A6261-1499V2	87	93	LuminalA	Luminal	Breast Tumor	Stratagene Human Universal Reference that contained 1/10 added MCF7 and ME16C RNAs
UB57		training	60	1	0	2	1	0	17	0	17	H1A6261-1500V2	91	94	LuminalA	Luminal	Breast Tumor	Stratagene Human Universal Reference that contained 1/10 added MCF7 and ME16C RNAs
UB58		training	58	1	1	2	1	0	19	0	19	H1A9529-0046C	80	95	LuminalA	Luminal	Breast Tumor	Stratagene Human Universal Reference that contained 1/10 added MCF7 and ME16C RNAs
UB60		training	72	0	1	3	2	0	20	0	20	H1A6433-2275_V2	1	96	HER2+/ER-	HER2+/ER	Breast Tumor	Stratagene Human Universal Reference that contained 1/10 added MCF7 and ME16C RNAs
UB61	pair 22	training	51	1	1	2	2	0	19	0	19	H1A9529-0065C	78	97	LuminalA	Luminal	Breast Tumor	Stratagene Human Universal Reference that contained 1/10 added MCF7 and ME16C RNAs
UB61-Int	pair 22	training										H1A7411-0107-C	77		LuminalA	Luminal	Breast Tumor	Stratagene Human Universal Reference that contained 1/10 added MCF7 and ME16C RNAs
UB62		training	28	1	1	1	1	0	9	0	9	H1A6149-021	132	98	NB	NB	Breast Tumor	Stratagene Human Universal Reference that contained 1/10 added MCF7 and ME16C RNAs
UB64		training	87	1	0	2	2	0	7	0	7	H1A6149-116	129	99	NB	NB	Breast Tumor	Stratagene Human Universal Reference that contained 1/10 added MCF7 and ME16C RNAs
UB66		training	88	1	0	1	2	0	9	0	9	H1A6989-8211-V2	112	100	LuminalA	Luminal	Breast Tumor	Stratagene Human Universal Reference that contained 1/10 added MCF7 and ME16C RNAs
UB67	replicate pair,	training	80	0	1	3	1	0	16	0	16	H1A9529-0044Na_C	46	101	Basal	Basal	Breast Tumor	Stratagene Human Universal Reference that contained 1/10 added MCF7 and ME16C RNAs
UB67-Repeat1	replicate pair,	training										H1A6730-745	47		Basal	Basal	Breast Tumor	Stratagene Human Universal Reference that contained 1/10 added MCF7 and ME16C RNAs
UB69		training	40	1	0	2	1	0	13	0	13	H1A7411-0108-C	82	102	LuminalA	Luminal	Breast Tumor	Stratagene Human Universal Reference that contained 1/10 added MCF7 and ME16C RNAs
UB71		training	60	0	0	3	1	0	7	0	7	H1A7411-0111-C	67	103	Basal	Basal	Breast Tumor	Stratagene Human Universal Reference that contained 1/10 added MCF7 and ME16C RNAs
UB78	pair 23	training			1							H1A6433-2276_V2	107	104	LuminalB	Luminal	Breast primary with an associated lymph node metastasis	Stratagene Human Universal Reference that contained 1/10 added MCF7 and ME16C RNAs
UB78-LN-Met	pair 23	training										H1A6433-2757_V2	106		LuminalB	Luminal	Lymph node metastasis	Stratagene Human Universal Reference that contained 1/10 added MCF7 and ME16C RNAs
UB79	pair 24	training	46	1	1	2	2	0	2	0	2	H1A6433-2758_V2	117	105	LuminalB	Luminal	Breast primary with an associated lymph node metastasis	Stratagene Human Universal Reference that contained 1/10 added MCF7 and ME16C RNAs
UB79-LN-Met	pair 24	training										H1A6433-2759_V2	116		LuminalB	Luminal	Lymph node metastasis	Stratagene Human Universal Reference that contained 1/10 added MCF7 and ME16C RNAs
