# Supplementary figures and images for: The molecular portraits of breast tumors are conserved across microarray platforms
Source: BMC Genomics. 2006 Apr 27;7:96. doi: 10.1186/1471-2164-7-96 (PMC1468408; doi:10.1186/1471-2164-7-96)

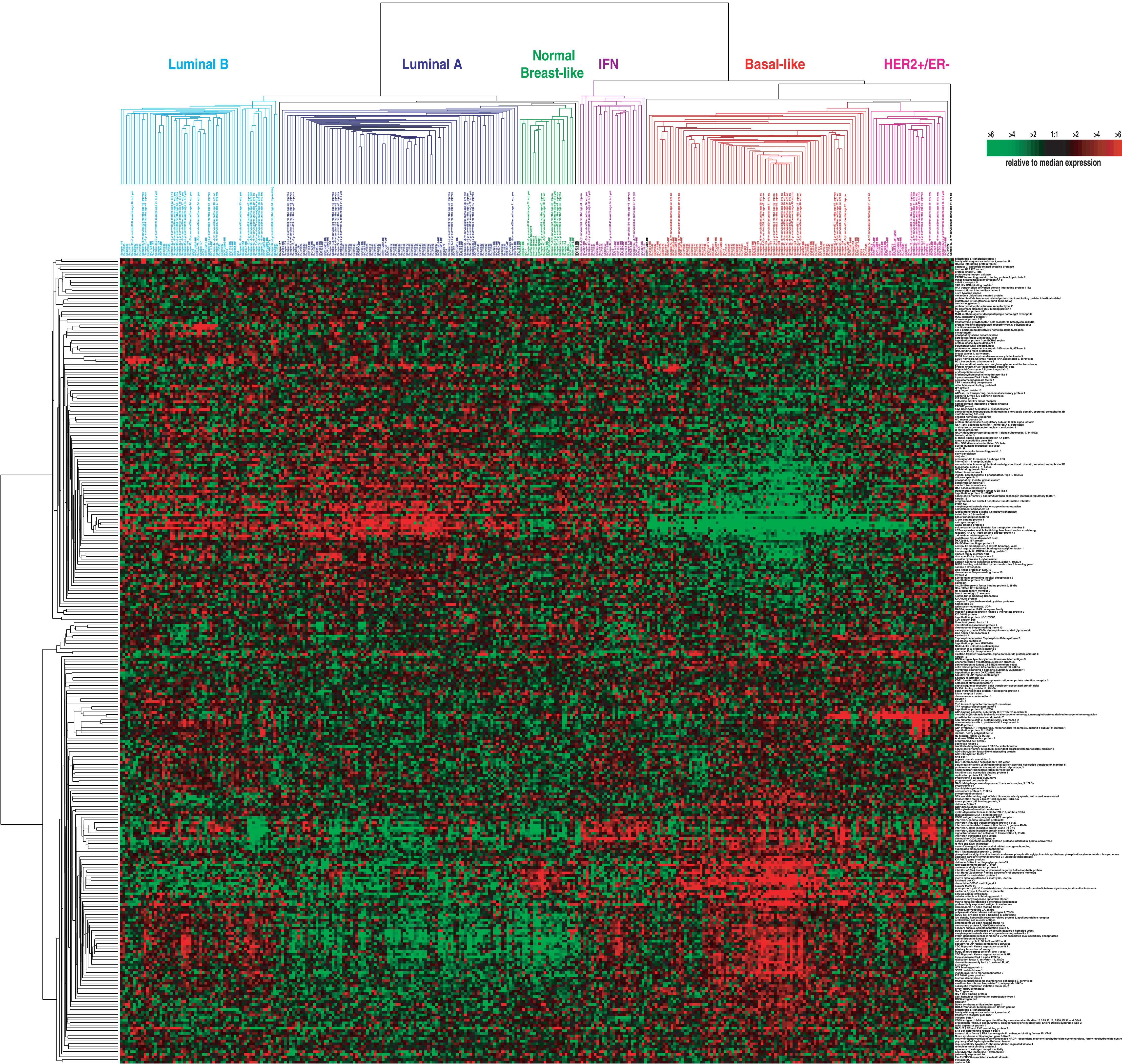

Supplement: Additional File 1 — Supplemental Figure 1. Complete hierarchical cluster diagram of the 315-sample combined test set analyzed using the Intrinsic/UNC gene set, which was reduced to 306 genes based upon the gene overlap between datasets. Sorlie et al. sample names begin with the letters "BC", Sotiriou et al. sample names begin with "Exp", and van't Veer et al. sample names begin with "sample". [file 1471-2164-7-96-S1.pdf]
